# Reviewing the Ultrasound Anatomy of the Gluteal Region and the Mapping of Fillers

**DOI:** 10.1111/jocd.70499

**Published:** 2025-10-15

**Authors:** Eliza Porciuncula Justo Ducati, Fabiano Nadson Magacho‐Vieira, Cláudia Borges Fontan Câmara, Gabriel de Sousa Lima, Camila Souza de Araujo Guarinello

**Affiliations:** ^1^ ED Ultrassonografia Dermatológica Sao Paulo Brazil; ^2^ Magacho Institute for Health Education; Batista Memorial Hospital Fortaleza Brazil; ^3^ Ultraplenna Ultrassonografia Especializada Clinic Sao Paulo Brazil; ^4^ Blanc Hospital Sao Paulo Brazil

**Keywords:** dermatological ultrasonography, gluteus, hyaluronic acid (HA) and gluteal mapping, hydrogel, polymethylmethacrylate (PMMA), silicone oil

## Abstract

**Introduction:**

The demand for buttock contouring and volumization has increased in recent decades, driven by growing interest in aesthetic procedures. However, a noticeable gap exists in the literature regarding the ultrasonographic anatomy of the gluteal region and the mapping of exogenous fillers. Despite the rising popularity of gluteal augmentation procedures, comprehensive studies focusing on the detailed visualization and characterization of these procedures using dermatological ultrasonography are scarce. This study aims to address this gap by thoroughly describing the ultrasonographic anatomy of the gluteal region and mapping the distribution of various fillers utilized.

**Objectives:**

This study aims to describe the ultrasonographic anatomy of the gluteal region and map the distribution of exogenous fillers.

**Methods:**

This descriptive study analyzed the ultrasonographic anatomy and filler mapping based on a cohort of 110 patients treated at a clinic in São Paulo, Brazil, from June 2023 to January 2025. Inclusion criteria were patients treated with a single or more types of filler and with or without complications. Examinations were performed using GE LOGIQ E ultrasound devices with probes ranging from 2 to 8 mHz, 3–15 mHz, and 3–22 mHz. Patients were positioned in the prone position, and the gluteal region was divided into four quadrants for systematic analysis, including the lumbar region, posterior thigh, trochanteric depression, and inguinal regions.

**Results:**

Ultrasound proved to be a non‐invasive, dynamic, sensitive, and accurate imaging technique for identifying the ultrasonographic characteristics of the main exogenous fillers in the gluteal region.

**Conclusions:**

The increasing demand for gluteal filling procedures underscores the importance of dermatological ultrasonography for differentiating and characterizing filler materials and enabling the early diagnosis of complications. The article proposes a systematic method for mapping the gluteal region using ultrasound; it advances the current knowledge of gluteal filler procedures by providing a detailed understanding of the ultrasonographic anatomy of the gluteal region. It highlights the role of dermatological ultrasonography in elucidating filler distribution and characteristics, paving the way for improved safety protocols and clinical practices in aesthetic procedures.

## 1 Introduction

The gluteal region, located between the trunk and the lower limbs, performs crucial physiological functions for the balance and movement of the human body. Furthermore, it is culturally associated with aesthetics and sexuality, often viewed as a symbol of physical beauty [1, 2]. Over the past few decades, the gluteal region has been the focus of various aesthetic procedures, reflecting a growing demand for modifications in contour and volume, with the goals of harmonizing and enhancing their appearance. With the increasing interest in aesthetic procedures, the utilization of ultrasonography techniques becomes essential to ensure safety and efficacy in performing these procedures, as well as to diagnose complications and assist in treatments. Ultrasonography allows for a detailed assessment of the gluteal anatomy and facilitates the identification of the characteristics of the fillers used, providing a safer approach.

Currently, there are several surgical and non‐surgical methods for enhancing the gluteal contours, including implants, autologous fat grafting (such as Brazilian butt lifts), collagen biostimulators (such as poly‐L‐lactic acid, calcium hydroxyapatite, polycaprolactone, among others), hyaluronic acid (HA)‐based fillers, polyacrylamide hydrogel (PAAG), and industrial liquid silicone [3, 4, 5]. These interventions range from more invasive surgical procedures to less invasive alternatives, each with varying degrees of complexity and risk. Surgical approaches, such as silicone implants and fat grafting, are widely used but carry risks such as infections, visible scarring, capsular contracture, and complications related to implant positioning [3, 5]. On the other hand, fillers like hyaluronic acid have gained popularity due to their lower invasiveness and, in some cases, the possibility of reversibility when the results are unsatisfactory [5]. However, methods like polyacrylamide hydrogel and industrial liquid silicone, while still used, have faced criticism and bans due to severe complications associated with their use [6, 7].

With the global increase in demand for aesthetic gluteal procedures, reflected by a 60.9% rise in demand for surgeries in 2023, according to the International Society of Aesthetic Plastic Surgery (ISAPS) [8], the evolution of these techniques necessitates a critical analysis of the available alternatives, their advantages, limitations, and risks to patient safety.

Ultrasound is a non‐invasive, dynamic, sensitive, easily accessible, and precise imaging technique for evaluating exogenous fillers applied to the gluteal region, although it is not yet part of the established protocols for these practices. The use of ultrasound enables the guidance of injectable filler procedures, pre‐ and post‐procedure assessment, and the monitoring and diagnosis of potential complications [4, 9, 10].

The integration of ultrasound technology into aesthetic procedures, particularly gluteal augmentation, offers significant advantages. One key benefit is the early diagnosis of complications associated with fillers, such as abscesses or granulomas, which may not be detectable through visual examination. Ultrasound also enhances the differentiation and characterization of various fillers, improving the understanding of their distribution within subcutaneous tissue, which aids in making informed clinical decisions [3, 5]. Additionally, the dynamic nature of ultrasound allows for real‐time visualization of gluteal structures and tissue responses to injected fillers, essential for assessing treatment efficacy [4]. Moreover, ultrasound provides precise mapping of filler distribution, crucial for ensuring the safety and effectiveness of procedures [9]. Overall, the use of ultrasound in evaluating gluteal fillers enhances safety and quality, leading to better patient outcomes while minimizing adverse effects.

## 2 Materials and Methods

This is a descriptive study of the ultrasonographic anatomy of the gluteal region and the ultrasonographic mapping of exogenous fillers. The technical descriptions and experience are based on the total number of gluteal mapping exams performed between June 2023 and January 2025, involving 110 patients. From this cohort, based on this sample, we detail a protocol for ultrasonographic gluteal mapping. Patients were treated at the ED Dermatological Ultrasonography clinic, located in the southern region of São Paulo, Brazil, between June 2023 and January 2025. Each patient underwent a distinct gluteal filler procedure or not prior to the examination, including the use of hyaluronic acid, autologous fat graft, polymethylmethacrylate (PMMA), hydrogel and silicone. All of the patients are female, with ages ranging from 20 to 65 years, and the average age is 35 years.

Inclusion criteria are patients who have undergone filling or not with only one or more types of filler and who do or do not present associated complications. Exclusion criteria are the presence of more than one type of gluteal filler and/or associated complications.

All patients signed an informed consent form for the use and publication of their images in the study, and all processes were conducted in accordance with the principles of the Declaration of Helsinki.

### 2.1 Operator Experience and Validation

All examinations were performed by the same operator, who has 7 years of training and experience in aesthetic patient imaging and dermatological ultrasound, with existing publications in the field. To ensure the validity of the findings and the examination method, the images from all gluteal mappings were shared with the co‐authors, who possess the same level of technical experience, for review and to compare any potential differences in image acquisition.

### 2.2 Standardized Gluteal Mapping Protocol

**FIGURE 1:**
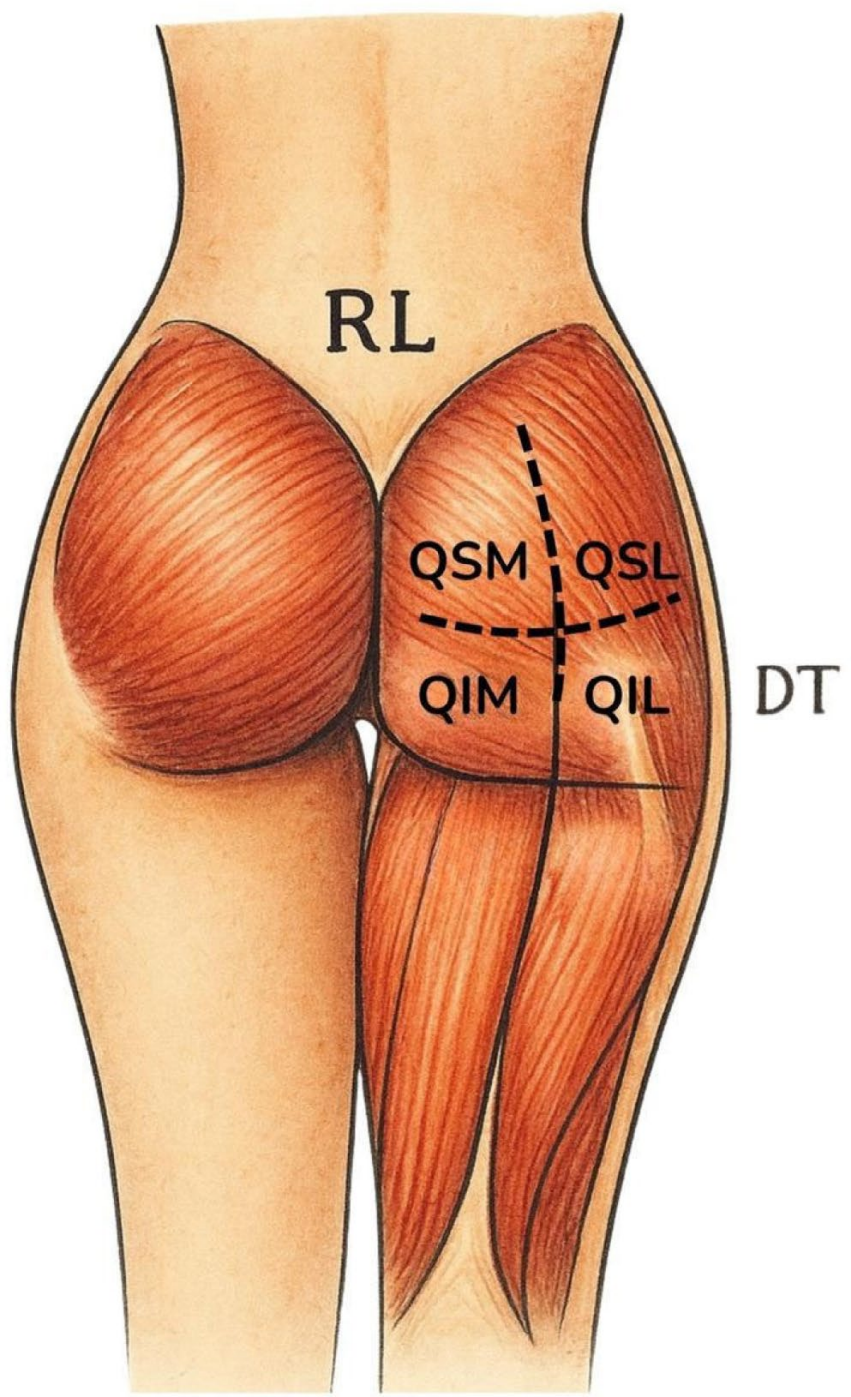
Topographical division of the gluteal region into four quadrants (created by the authors).

The examinations were performed using GE brand devices, models LOGIQ E, with patients in the prone position. A standardized acquisition protocol was followed for each patient. First, both buttocks were divided into four quadrants for topographic analysis: superolateral (SLQ), superomedial (SMQ), inferolateral (ILQ), and inferomedial (IMQ). Figure 1 illustrates the topographical division of the gluteal region into four quadrants, as described, facilitating systematic ultrasonographic analysis.

Adjacent areas, including the junction of the quadrants (JQ), trochanteric depression, posterior thigh, and lumbar region, were also systematically examined due to the possibility of filler presence or migration. The approach utilized multiple transducers in sequence. The examination began with a mid‐frequency linear transducer (3–15 MHz) for a complete scan of both superficial and deep regions. Next, a low‐frequency convex transducer (2–8 MHz) was used to analyze the deep musculature and vascularization in detail. Finally, a high‐frequency linear transducer (3–22 MHz) allowed for a detailed analysis of the superficial subcutaneous tissue and dermis to characterize products with greater morphological and vascular specificity. All findings were analyzed in both longitudinal and transverse axes, with images acquired in both planes and, when necessary, comparative images. In cases with findings suggestive of industrial silicone, the examination was extended to the inguinal regions to search for product infiltration in the lymph nodes and in distant areas. The minimum number of images was 20–25 for a normal mapping, increasing according to the number of findings to be documented.

### 2.3 Data Analysis

The study provides detailed qualitative descriptions of the findings. In addition, all measurable findings identified during the gluteal mapping were systematically recorded with quantitative data. This included measurements in all axes, volume calculation (when applicable), and the distance from the skin or important adjacent structures. For example, a deposit of hyaluronic acid in the subcutaneous tissue would be described quantitatively as: “an oval‐shaped anechoic deposit with thin walls and posterior acoustic enhancement, measuring 0.5 × 0.4 × 0.8 cm (volume: 0.16 cm^3^), located at a distance of 0.3 cm from the skin.”

## 3 Results

In recent years, the demand for aesthetic procedures in the gluteal region has increased considerably. This growth has highlighted the need to standardize and establish techniques for the dermatological ultrasonographic study of this body area. In this context, the aim of this study is to present the ultrasonographic and superficial anatomy of the gluteal region and the ultrasonographic characterization of the main types of fillers currently used.

Dermatological ultrasound of the gluteal region can be obtained using multifrequency linear ultrasound transducers—which operate at high frequency (6–15 MHz) for analyzing superficial layers, such as the skin and subcutaneous tissue—and convex transducers—which operate at low frequency (2–8 MHz) for analyzing deeper layers, such as muscle, vascular, and bony structures. The ultrasound of the gluteal region can be performed by dividing it into four quadrants—two upper (medial and lateral) and two lower (medial and lateral)—and marking the junctions of these quadrants.

The primary area of risk in gluteal fillers is located in the medial quadrants, adjacent to the intergluteal cleft, where the major vascular and nervous structures of the region are located, such as the superior and inferior gluteal arteries and veins, as well as the sciatic nerve.

The ultrasonographic analysis of the gluteal mapping should begin with linear transducers to characterize the superficial layers, especially the subcutaneous layer, where most exogenous fillers are injected. Next, deeper regions are examined using low‐frequency convex transducers (2–8 MHz) to detail both the musculature and the major vessels and nerves in this area.

The ultrasonographic anatomy in gluteal mapping involves the analysis of the following body structures, from superficial to deep: skin—epidermis and dermis—subcutaneous tissue (areolar and lamellar layers), separated by the superficial fascia, deep muscular layers, including the muscular fascia and the gluteus maximus, gluteus medius, and piriformis muscles, where the main anatomical structures are located, including the superior and inferior gluteal arteries, veins, and sciatic nerve, followed by the bony layer, vascular‐nervous bundle, and bones.

The skin, composed of the epidermis and dermis, is the first ultrasonographic layer to be analyzed in the gluteal region, where bio‐stimulators such as poly‐L‐lactic acid and calcium hydroxyapatite are often deposited. The characterization of this layer is performed using linear transducers with frequencies ranging from 3 to 22 MHz.

Below the skin lies the subcutaneous tissue, composed of two layers: the superficial, called areolar, and the deep, called lamellar. This topography, mainly made up of fat and ligaments, typically contains most of the exogenous gluteal fillers, such as hyaluronic acid, autologous fat, silicone, polyacrylamide gel, and polymethylmethacrylate.

After a detailed analysis of the subcutaneous layer, the transducer should be changed to a convex one for studying the deep muscular fascia and the gluteus maximus muscle, which overlays the subsequent layer, where the gluteus medius and piriformis muscles, the gluteal vessels, and the sciatic nerve are located. Exogenous gluteal fillers may be located in either the superficial or deep subcutaneous layer or the intramuscular layer.

The musculature of the gluteal region is composed, superficially, of the gluteus maximus, followed by a deeper layer that includes the gluteus medius and piriformis muscles, the sciatic nerve, and the superior and inferior gluteal vessels. The trochanteric depression of the lateral gluteal region is formed by the “contrast” between the hypertrophy of the gluteus maximus and medius muscles and the lateral fibrous tendinous tissue of the gluteal aponeurosis, which maintains the same volume, particularly in patients undergoing hypertrophy training, causing this lateral depression in the gluteus.

**FIGURE 2:**
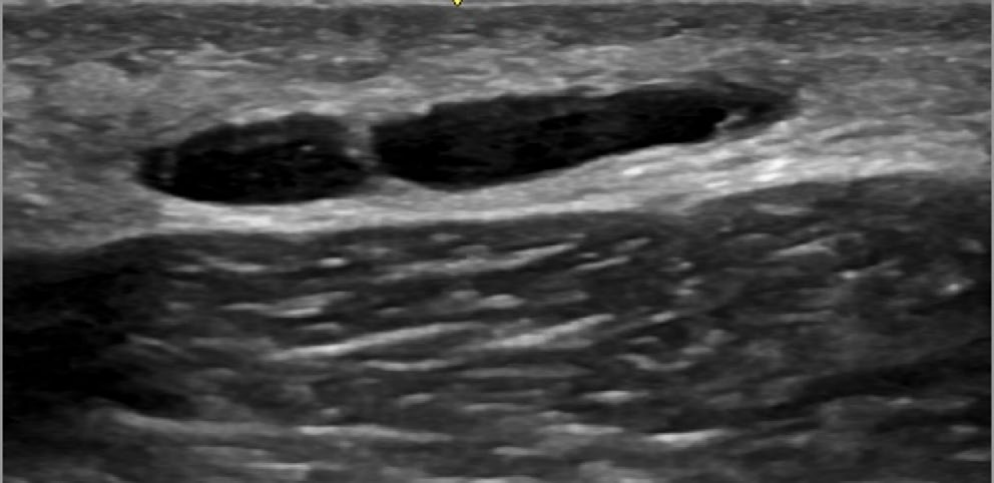
Hyaluronic acid. Ultrasound image showing an elongated anechoic deposit with thin walls in the subcutaneous layer of the gluteal region, adjacent to the muscular fascia. Characteristic of hyaluronic acid (HA) filler.

Hyaluronic acid is a biodegradable filler typically injected into the superficial or deep subcutaneous layers of the upper gluteal quadrants and at the junction of these quadrants, with the aim of achieving superior and posterior projection of the gluteal region. The ultrasonographic appearance is characterized by anechoic vesicles with thin walls, free of internal debris, elongated or rounded, sometimes exhibiting posterior acoustic enhancement (Figure 2).

**FIGURE 3:**
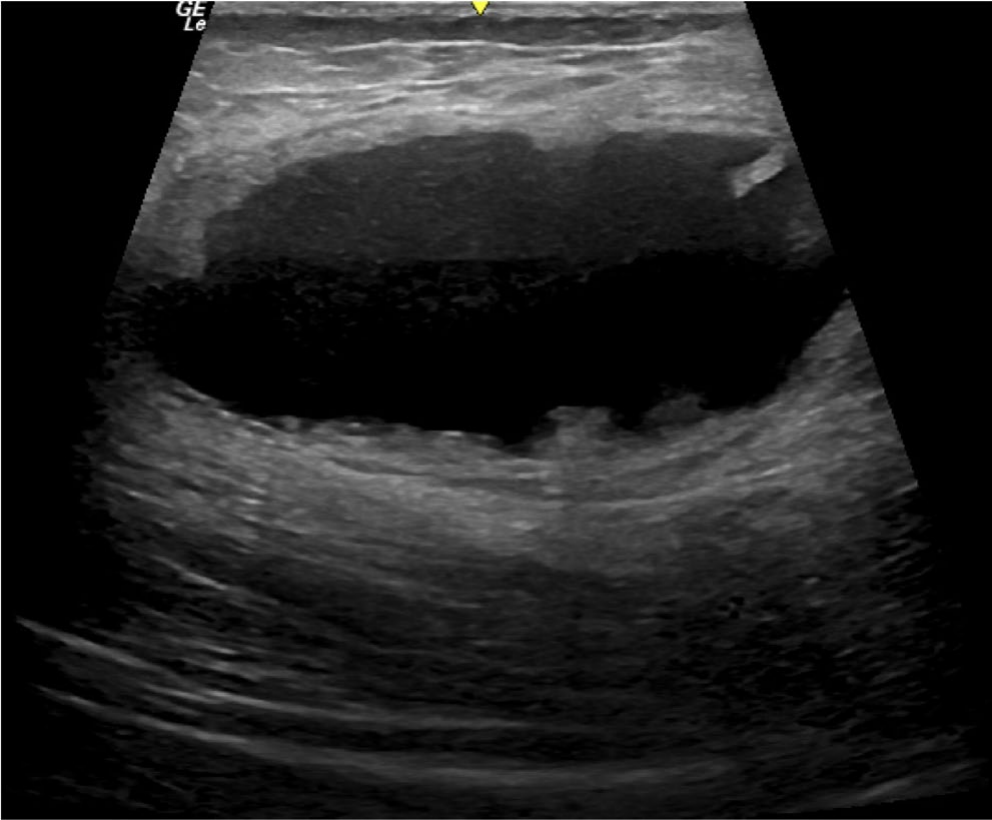
Post‐grafting fat collection. Ultrasound image of a post‐grafting fat collection with thick walls and an anechoic center, typically observed after autologous fat grafting with a complication known as central necrosis.

Autologous fat grafting is commonly associated with plastic surgery procedures, where fat is harvested during liposuction of the abdomen and flanks, followed by fat processing and grafting into the gluteal region. Typically, fat deposits appear as round or elongated hypoechoic formations, well defined, without associated artifacts. Depending on the fat injection technique, which is usually performed retrogradely with a cannula and in small boluses in the subcutaneous layer, these deposits are seen on ultrasound as being positioned in a linear fashion. However, at times, they deviate from this pattern and are found randomly distributed across the gluteal regions (Figure 3).

**FIGURE 4:**
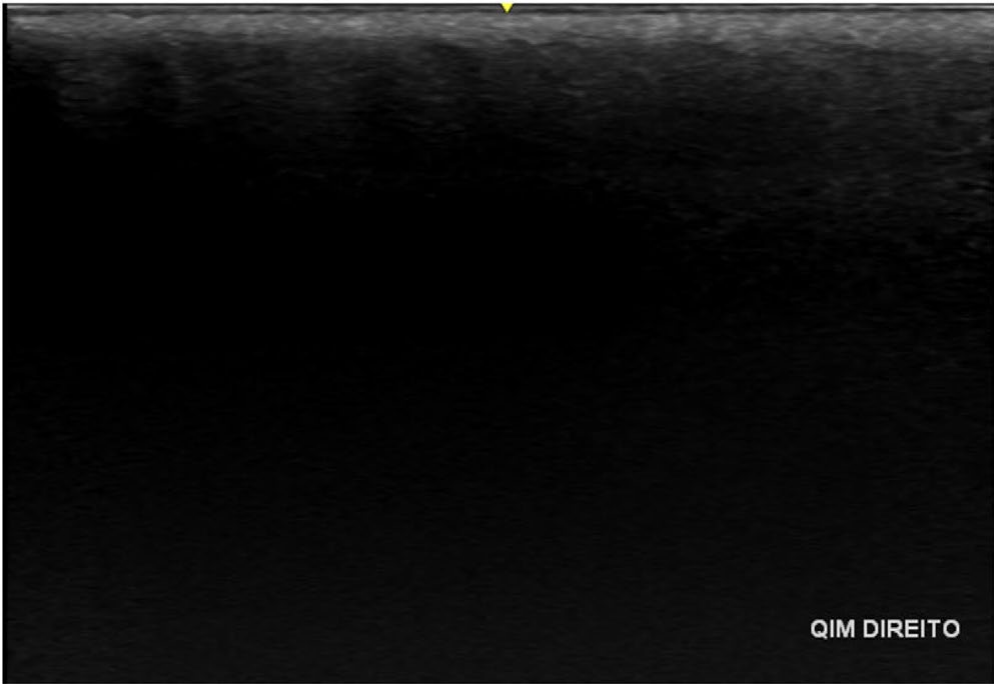
First silicone oil. Ultrasound images showing infiltrative echogenic silicone oil in the epidermis, producing a characteristic “snowstorm” artifact. Anechoic vesicles, taller than wide, are interspersed within the filler, with a posterior echogenic linear artifact indicative of pure silicone.

**FIGURE 5:**
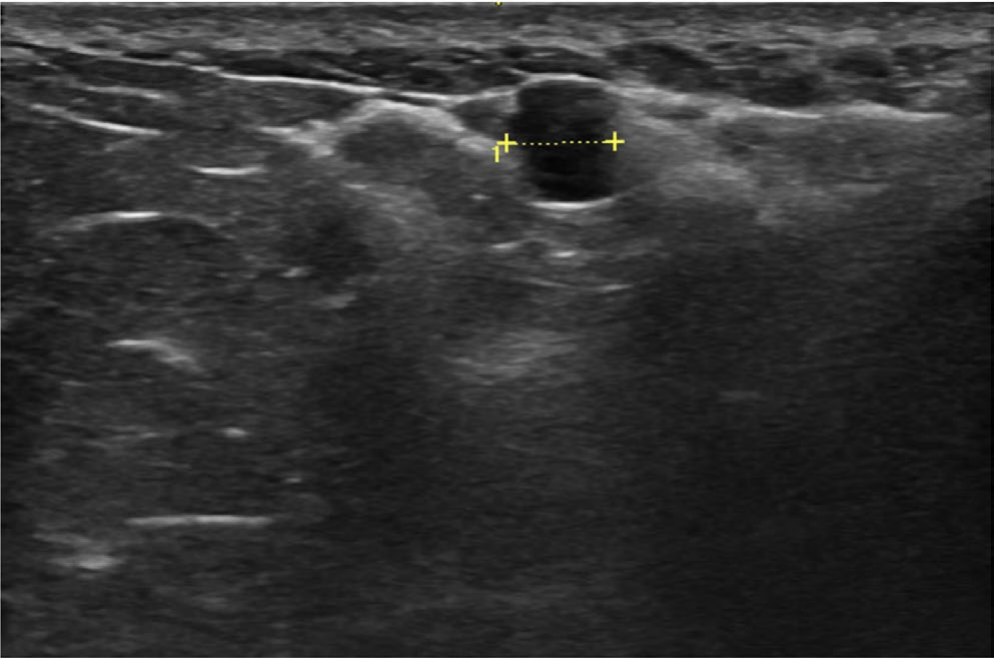
Liquid silicone. Ultrasound images showing infiltrative echogenic silicone oil in the epidermis, producing a characteristic “snowstorm” artifact. Anechoic vesicles, taller than wide, are interspersed within the filler, with a posterior echogenic linear artifact indicative of pure silicone.

It is important to emphasize that, although fat is a “natural” filler, seeing as it is harvested from the patient's own body, it also carries risks of complications. This is because the success of the procedure depends on the injection technique used and its interaction with other fillers that may already be present in the region. The main complications associated with fat grafting are deposits of fat necrosis, which can appear as collections or calcifications, sometimes palpable or even visible. Although industrial silicone is not permitted for use as a body injectable, there are still cases of patients who have undergone gluteal filling with this permanent material. On ultrasound, silicone appears as a classic hyperechoic structure, which tends to migrate towards the epidermis in an infiltrative manner, causing an intense “dirty” shadow artifact with a “snow storm” appearance, which prevents the visualization and study of structures posterior to the filler (Figure 4). When examining the ultrasonographic finding of silicone oil, it is common to identify the presence of anechoic vesicles, with greater height than width, accompanied by a posterior linear echogenic artifact, indicating the presence of pure silicone vesicles. Often, when injected deeply into the muscle, this oily filler superficializes towards the muscular fascia, presenting the same ultrasonographic characteristics (Figure 5).

The main complication of silicone is related to its easy spreadability through the planes and at a distance from the injection site, making it mandatory to conduct complementary studies of the lumbar region, thighs, anterior and posterior legs, and inguinal areas to investigate possible lymph node infiltration in these cases.

**FIGURE 6:**
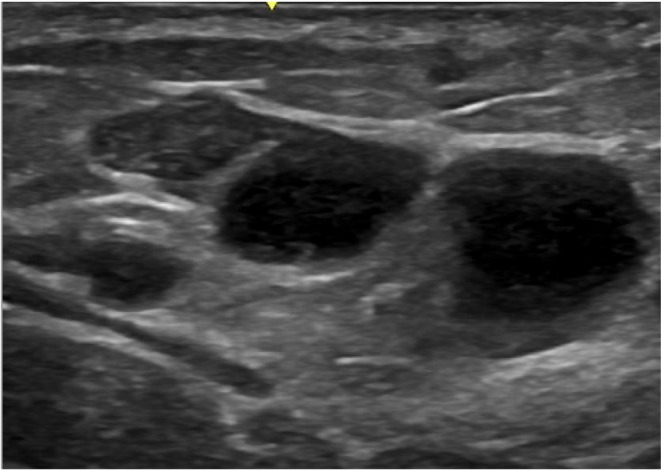
First polyacrylamide gel. Ultrasound image of polyacrylamide gel (PAAG) in the gluteal region, showing conglomerated hypoechoic pseudocystic areas with internal hyperechoic deposits, occasionally adjacent to anechoic vesicles.

Polyacrylamide gel, also known as polyacrylamide hydrogel or simply PAAG, was widely used in the gluteal region between 2000 and 2010 when its use for aesthetic purposes was permitted. Therefore, this material is still found in patients during ultrasonographic mapping of the gluteal area. This filler can be characterized in the gluteal region as well‐defined, anechoic vesicles, either singular or grouped, sometimes filled with internal debris (Figure 6).

**FIGURE 7:**
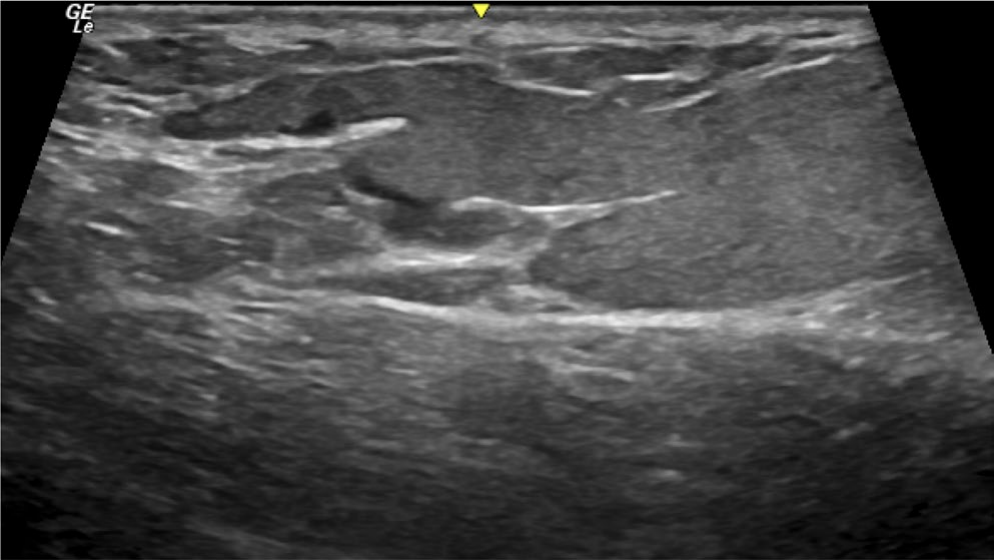
Second polyacrylamide gel. Ultrasound image showing well‐defined hypoechoic collections with internal debris, which may exhibit movement during static maneuvers. Characteristic of PAAG migration and potential complications.

Hypoechoic and heterogeneous collections, well or poorly defined, elongated along the axis parallel to the skin, are commonly located in the superficial or deep subcutaneous plane. These collections also represent one of the forms found in patients with polyacrylamide gel or hydrogel in the gluteal region (Figure 7). Polyacrylamide gel has the ability to migrate to areas distant from its injection site, such as the lumbar region and thighs. Due to its gelatinous/liquid content, it can serve as a culture medium for skin germs, leading to abscesses and infected collections, which may occasionally drain through fistulas in the skin.

Polymethylmethacrylate, or PMMA, is a permanent filler used for the correction of body asymmetries and has been widely applied in the gluteal regions in either the deep intramuscular or superficial subcutaneous planes for volumization. The newer generations of products containing polymethylmethacrylate have varying concentrations, with options of 5%, 10%, and 15%, most commonly used for subcutaneous tissue injection, and 30%, typically used for intramuscular injection.

**FIGURE 8:**
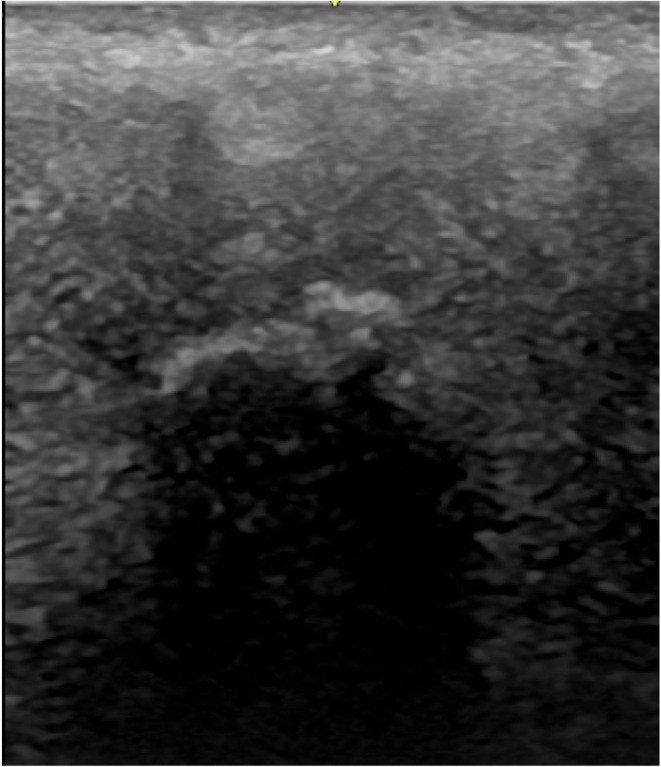
Polymethylmethacrylate (PMMA) in the subcutaneous tissue of the gluteal region. Hyperechoic formation with multiple irregular deposits conglomerated together, producing a posterior acoustic shadowing artifact. The formation consists of tiny hyperechoic foci with a “comet‐tail” artifact, a feature typically associated with this material (PMMA). This nodular formation is located within the subcutaneous layer, a few millimeters beneath the most superficial layers of the gluteal skin.

**FIGURE 9:**
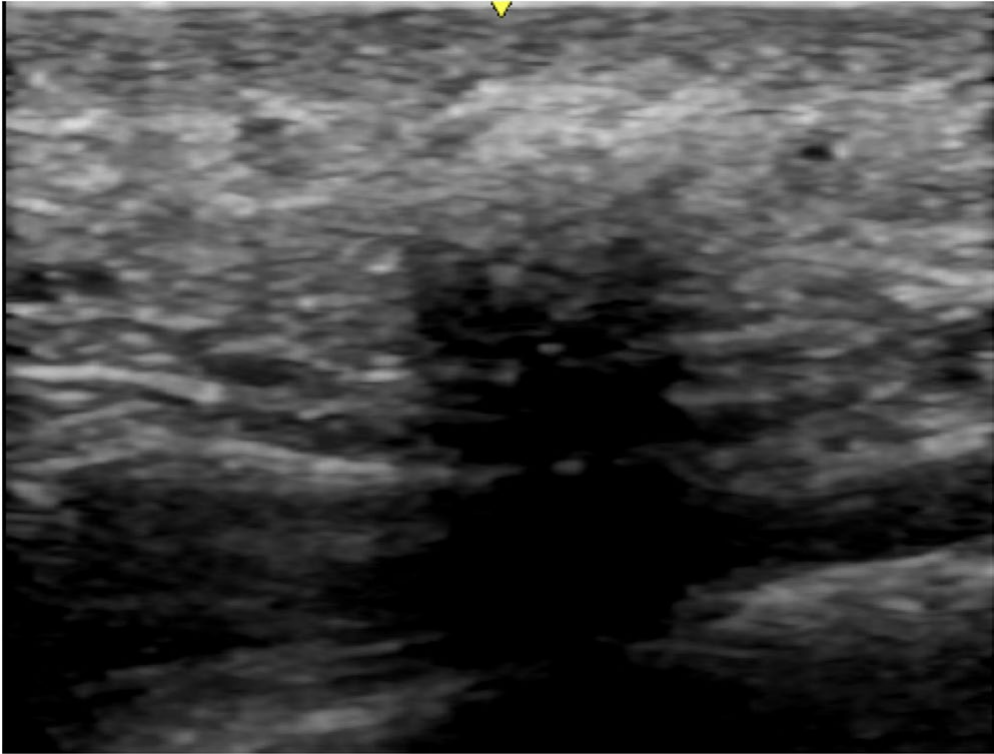
Ultrasound image of polymethylmethacrylate (PMMA) in the gluteal region. Subtle hyperechoic formation composed of multiple tiny hyperechoic deposits with a “comet‐tail” artifact, more clearly visualized using high‐frequency probes. This nodular formation is located very close to the superficial layers of the dermis and produces a posterior acoustic shadowing artifact. Note how this accumulation of PMMA is more discreet and presents a less hyperechoic surface compared to the nodule shown in Figure 8.

The ultrasonographic characteristics of polymethylmethacrylate in the gluteal region include poorly defined hyperechoic formations with a spiculated appearance, punctate hyperechoic deposits with a “comet‐tail” artifact, as well as a posterior acoustic shadow artifact. This appearance is found when the product is injected into the subcutaneous tissue (Figures 8 and 9).

**FIGURE 10:**
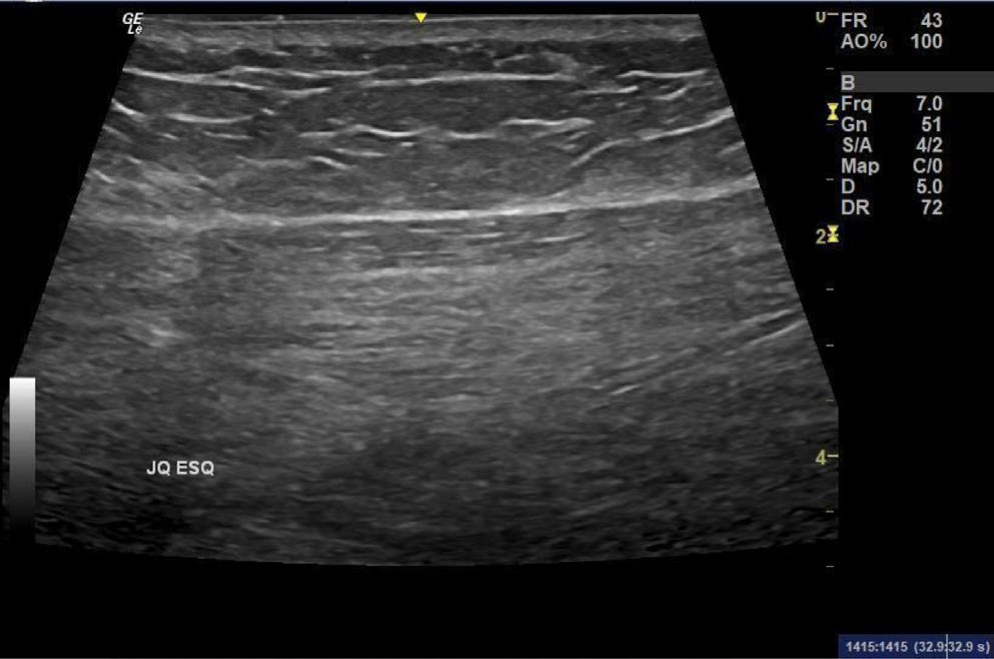
Third polymethylmethacrylate. Ultrasound image (10) showing increased echogenicity and heterogeneity of muscle fibers in the gluteus maximus, characteristic of PMMA injection into the muscular plane.

Another important point to highlight is that polymethylmethacrylate, when injected into the muscular planes, may exhibit increased echogenicity and loss of the characteristic fibrillar pattern of the muscle (Figure 10).

The complications associated with polymethylmethacrylate most commonly reported on ultrasound of the gluteal region include nodules in the subcutaneous tissue, which may be palpable and even visible.

## 4 Discussion

The procedures for gluteal filling have grown significantly in recent years, accompanied by an increase in complications associated with them. This underscores the importance of dermatological ultrasound, both for the differentiation and characterization of the materials used and for the early diagnosis of complications. The present study on the ultrasonographic anatomy of the gluteal region and the mapping of dermal fillers reveals findings that are highly consistent with the existing literature, underscoring the growing role of ultrasonography as a vital tool in clinical aesthetic practice. The results highlight the necessity of employing high‐ and medium‐frequency linear transducers for assessing superficial soft tissues, while low‐frequency convex transducers are more appropriate for deeper anatomical structures. This methodology is supported by prior studies, such as those by Sigrist et al. [4] and Lopes et al. [9], which emphasize the value of ultrasonography for real‐time, dynamic assessment of filler behavior within tissue planes—an approach reaffirmed by the current investigation.

A notable observation in this study is the marked increase in complications related to gluteal augmentation procedures, reinforcing the need for early detection and diagnosis through imaging. Ultrasonographic evaluation emerges as a non‐invasive, cost‐effective, and accessible modality for identifying adverse events, such as filler migration or inflammatory responses. These findings align with those of Mello et al. [11] and Mendes et al. [6], who describe complications particularly associated with the use of industrial silicone and advocate for early intervention using imaging‐based strategies.

Beyond diagnostics, gluteal mapping provides critical information that directly influences therapeutic planning. The examination is used to diagnose existing products and their possible associated complications. The practical applications of this are significant. For instance, a patient suspecting a PMMA injection and planning a surgical removal may discover through ultrasound that the product is actually silicone with migration to the lumbar region. This finding would completely redirect the surgical approach, as the technique for removing liquid/gel‐like silicone differs entirely from that for solid PMMA, which is enmeshed with tissue. In another scenario, for a patient with nodular formations who wishes to undergo new harmonization procedures, the precise description of the product type and its exact location will guide the physician in choosing the correct plane for the new injections, thereby enhancing safety and efficacy.

The study also contributes to the literature by mapping various commonly used filler substances—namely hyaluronic acid, autologous fat, polymethyl methacrylate (PMMA), hydrogel, and silicone—within the gluteal region. The characterization and identification of these materials via ultrasonography are in accordance with reviews by Valente et al. [5] and Oranges et al. [2], who emphasize that both the choice of filler and its accurate localization under ultrasound guidance are critical to procedural safety and effectiveness. This reinforces the importance of a standardized and methodical approach when working with injectable fillers in high‐risk anatomical areas. Moreover, the study advocates for the development and adoption of standardized ultrasonographic protocols for gluteal procedures, alongside targeted training for healthcare professionals. This recommendation reflects current aesthetic medicine guidelines, as discussed by Dai et al. [12] and Ibrahim et al. [13], which underscore the importance of continuous professional development and the integration of advanced imaging technologies in routine practice. By minimizing procedural risks and enhancing treatment outcomes, such training initiatives are essential for maintaining high standards in aesthetic interventions.

In summary, the findings of this study contribute meaningfully to the understanding of gluteal anatomy through ultrasonography, while also addressing the critical need for early complication detection, appropriate filler mapping, and professional training. These insights bridge the gap between theoretical knowledge and clinical application, reinforcing the role of ultrasonography as a cornerstone in contemporary aesthetic practice.

For the ultrasonographic mapping examination of the gluteal region should be performed using specific high‐ and medium‐frequency transducers—indicated for the analysis of the subcutaneous tissue, where most fillers in this region are located—and a low‐frequency convex transducer—for a detailed study of the deep muscular and vascular‐nervous regions.

The study's main limitations consisted of all participants being female and without complications, introducing selection bias. Variability in examination timing may affect consistency, and lack of comparison with other imaging methods reduces robustness.

Future research should focus on larger, multicenter studies to validate ultrasonographic techniques for different fillers. Developing standardized imaging protocols and training programs could improve accuracy and safety. Additionally, exploring the long‐term evolution of various fillers with ultrasound imaging may aid in early complication detection and management.

## Author Contributions

Eliza Porciuncula Justo Ducati contributed to the search for scientific articles, the writing of this article, the review of the content and the suggestion of revisions, the conduct and analysis of imaging examinations, and the description of ultrasonographic findings. Fabiano Nadson Magacho‐Vieira contributed her expertise by reviewing the content and suggesting revisions. Cláudia Borges Fontan Câmara contributed her expertise by reviewing the content and suggesting revisions. Gabriel de Sousa Lima contributed to the search for scientific articles and Fabiano Nadson Magacho‐Vieira contributed her expertise by reviewing the content and suggesting revisions. Cláudia Borges Fontan Câmara contributed her expertise by reviewing the content and suggesting revisions. Gabriel de Sousa Lima contributed to the search for scientific articles and Fabiano Nadson Magacho‐Vieira contributed her expertise by reviewing the content and suggesting revisions. Cláudia Borges Fontan Câmara contributed her expertise by reviewing the content and suggesting revisions. Gabriel de Sousa Lima contributed to the search for scientific articles and the writing of this article. Camila Souza de Araujo Guarinello contributed to the search for scientific articles and the writing of this article.

## Ethics Statement

This study was approved by the Research Ethics Committee of Blanc Hospital through Plataforma Brasil (approval number: CAAE 85749825.0.0000.0398).

## Conflicts of Interest

The authors declare no conflicts of interest.

## Data Availability

Research data are not shared.
